# Translocation of Threatened New Zealand Falcons to Vineyards Increases Nest Attendance, Brooding and Feeding Rates

**DOI:** 10.1371/journal.pone.0038679

**Published:** 2012-06-14

**Authors:** Sara M. Kross, Jason M. Tylianakis, Ximena J. Nelson

**Affiliations:** School of Biological Sciences, University of Canterbury, Christchurch, New Zealand; Macquarie University, Australia

## Abstract

Anthropogenic landscapes can be rich in resources, and may in some cases provide potential habitat for species whose natural habitat has declined. We used remote videography to assess whether reintroducing individuals of the threatened New Zealand falcon *Falco novaeseelandiae* into a highly modified agricultural habitat affected the feeding rates of breeding falcons or related breeding behavior such as nest attendance and brooding rates. Over 2,800 recording hours of footage were used to compare the behavior of falcons living in six natural nests (in unmanaged, hilly terrain between 4 km and 20 km from the nearest vineyard), with that of four breeding falcon pairs that had been transported into vineyards and nested within 500 m of the nearest vineyard. Falcons in vineyard nests had higher feeding rates, higher nest attendance, and higher brooding rates. As chick age increased, parents in vineyard nests fed chicks a greater amount of total prey and larger prey items on average than did parents in hill nests. Parents with larger broods brought in larger prey items and a greater total sum of prey biomass. Nevertheless, chicks in nests containing siblings received less daily biomass per individual than single chicks. Some of these results can be attributed to the supplementary feeding of falcons in vineyards. However, even after removing supplementary food from our analysis, falcons in vineyards still fed larger prey items to chicks than did parents in hill nests, suggesting that the anthropogenic habitat may be a viable source of quality food. Although agricultural regions globally are rarely associated with raptor conservation, these results suggest that translocating New Zealand falcons into vineyards has potential for the conservation of this species.

## Introduction

Agricultural expansion and intensification is a principal contributor to habitat change [1] and represents the “greatest extinction threat to birds" [2]. Raptor species worldwide have suffered declines [3], largely as a result of anthropogenic activities linked with agriculture, such as land clearing and the use of poisons for pest control [4]. Additional causes of mortality include persecution as a result of human-wildlife conflict and electrocution on electro-utility structures [5]–[7].

Raptor declines can be mitigated through the reintroduction of individual birds from their strongholds in order to bring threatened species back to their historic ranges [8]. Reintroductions have been successfully used to expand the ranges of a number of threatened raptors worldwide [9]–[10]. However, release sites for reintroduction programs normally comprise regions of natural habitat from which raptors have become extirpated. With land increasingly being put to use for anthropogenic purposes, there is inevitably a conflict when land is set aside for conservation. Consequently, there have been calls for increasing biodiversity conservation outside of the traditional reserve system [11]. Conservation efforts could be considered within primary production systems [12]–[13] by using farming practices that are more wildlife-friendly [2], provided that the species in question can survive within such agricultural landscapes.

There is extensive variability in how well raptors adjust to human landscapes, with some species being unable to inhabit modified habitats while others show considerable flexibility in this regard [14]. Conservation scientists have traditionally been slow to incorporate animal behavior when developing sustainable conservation management plans and policy [15]–[16], and this lack of consideration of the behavior of the animal in question has sometimes resulted in failed reintroductions [17]. As the ability of translocated individuals to display adaptive behavior in novel environments can influence the success of reintroduction projects, it should be examined closely at the onset of a reintroduction [8], [15]–[18]. This need to assess the behavioral ramifications of translocation is particularly acute when animals are reintroduced into anthropogenic landscapes. In these landscapes, translocated individuals must be able to forage, find shelter, and reproduce in order for a reintroduction program to succeed [9], [17]–[18].

In Marlborough, New Zealand’s largest wine-growing region, there is an intensive monoculture of vineyards spread throughout the valleys that were once inhabited by the now threatened New Zealand falcon, *Falco novaeseelandiae*, the country’s only remaining endemic bird of prey [19]. To combat the decline of falcons in Marlborough a project called ‘Falcons For Grapes’ (FFG) was established in 2005 to reintroduce falcons into the vineyard-dominated valleys of the region [20], [21]. As its name suggests, this project aims to use falcons to benefit the wine industry through their release into vineyards, while at the same time benefitting falcons through access to higher prey densities in vineyards and an expansion of their range [21]. Recent work has shown that falcon presence in vineyards is associated with considerable economic savings through a reduction in grape damage caused by passerine birds [22]. However, whether there is a simultaneous benefit to the falcon population is, as yet, unknown. Although vineyards have high densities of potential vertebrate prey (particularly European birds), falcons relocated to vineyards are also enticed to stay through supplementary feeding schemes.

The FFG project presented us with a unique opportunity to do a comparative analysis of the breeding behavior of reintroduced falcons in vineyards with falcons found in the nearby hills. We use these comparative data to compare the chick-rearing behavior and ability of falcons reintroduced into vineyards with that of falcons breeding naturally within the surrounding hill habitats. Falcon chicks hatch at roughly 31 g and reach full adult weight (330 g for males, and 531 g for females) in a 35-day rearing period [23]. This necessitates that adult falcons provision chicks with a large amount of prey each day. Feeding rates during the chick-rearing period dictate chick survival and contribute heavily to breeding success rates and population trends [24]. We therefore focused our study on comparing the food provisioning rates and the biomass of prey items delivered to falcons in both the vineyards and hills.

Generally, raptor species share biparental care duties during incubation and when their altricial chicks first hatch [24]. Extrinsic factors, such as habitat quality and prey abundance, may influence the time budget allocated by raptors to different activities and thus potentially affect breeding success [25]. Parents must balance the need to feed their young against the increased exposure of young to potential nest predation while their parents are foraging. New Zealand falcons nest in scrapes on the ground, and their nests are vulnerable to high levels of predation, mainly by introduced mammals such as feral cats (*Felis felis*) and stoats (*Mustela erminea*) [Kross SM, Tylianakis JM, Nelson XJ unpublished manuscript]. Areas of high prey density may therefore benefit falcons considerably through a reduction of time spent searching for prey, with a concomitant increase in nest attendance rates which may be associated with higher nesting success, as found in peregrine falcons (*Falco peregrinus*) [25].

Here, we provide evidence of the impact of anthropogenic habitat on prey provisioning rates, parental nest attendance, and brooding rates at nests of the threatened New Zealand falcons. By examining how the parental behavior of the New Zealand falcon differs between hill and anthropogenic vineyard habitats, we provide further evidence that behavioral studies should be inextricably tied to the implementation of sustainable conservation management plans.

## Methods

### Ethics Statement

This research was conducted according to relevant national and international ethics guidelines and permits were provided by the University of Canterbury (2008/27R) and the New Zealand Department of Conservation (NM-23677-FAU).

### Study Species

Despite its threatened status, little is known about the breeding behavior of the New Zealand falcon. New Zealand falcons evolved in the absence of land-dwelling mammals, and therefore lack the morphological and behavioral adaptations necessary to deal with mammalian predators [26]. For example, they often nest in ‘scrapes’ on the ground, making them prone to high levels of nest predation [23], [27]–[28]. In the New Zealand falcon, incubation lasts for 30 days, followed by a 30–35 day rearing period during which chicks develop the ability to thermoregulate (at approximately 12 days), reach full adult weight (at approximately 20 days), and develop feathers. Adult females undertake the majority of nest attendance, nest defense, and feeding of chicks, while male falcons assume most of the foraging and provision females and chicks with food [23]. As chicks grow, female falcons begin to take part in foraging and food provisioning [23].

Falcon nests were located by interviewing local farmers and forestry workers. Non-vineyard falcon nests (‘hill nests’) were found either in hillside forestry plantations (*Pinus radiata*) or in steep-sided valleys dominated by a mix of native and introduced grasses and dense scrub [28]. In contrast, vineyard falcon nests (‘vineyard nests’) were near the valley floor, usually within a vineyard, although on one occasion, within a forestry plantation adjacent to a vineyard. The key differences between the nest types were that vineyard adults were manipulated by the FFG project, whereas hill adults were not manipulated. Vineyard adults had been translocated into the vineyards as juveniles, were offered supplementary food on a daily basis (one-day-old poultry chicks), and had their nests raised from the ground into artificial nests in order to reduce the chances of predation by invasive mammals. Over 50 falcons were released by the FFG project in the valleys of Marlborough between 2005 and 2011, and eight have been confirmed to breed within the vineyard region, including the four vineyard nests that we monitored for this study (R. Seaton, pers. comm.).

### Data Collection

Our data were based on footage obtained from six hill nests (101 days or 1473 recording hours) and four vineyard nests (88 days or 1333 recording hours) monitored between 2008 and 2011. We were only able to monitor five of the eight confirmed breeding falcons that were released as part of the FFG project because the remaining nesting events were before our study period, were outside of the vineyard region, or failed before we could monitor them. We used a portable remote videography system with a near-infrared camera placed at the edge of the nest or mounted to the side of nest barrels in the case of vineyard nests. The system was set to record (at 30 fps) based on a motion-detection threshold of 10–15%, and has been shown to lose only 16% of potential recording hours, primarily due to battery failure or camera dislodgement [28]. For these data, if over 50% of recording hours in any given day were missed, that day was excluded from the dataset. Video was reviewed using Quick-Time Player (version 7.6.4; Apple Inc, Cupertino, CA, USA) at a maximum speed of four times normal speed to a minimum speed of frame-by-frame, allowing quick review of non-important files and detailed review of important events, such as feeding.

Monitored nests during the chick rearing stage had 1, 2, or 3 chicks. The number of chicks in these nests did not differ significantly between hill (*n* = 13) and vineyard (*n* = 8) nests (Mann Whitney U = 12.0, P = 0.91; for both habitats median  = 2.0; 1^st^ and 3^rd^ quartiles are 1.0 and 3.0). In the rare (i.e. <10% of recordings) cases where one or more of the chicks had moved outside of the recording area, we stipulated that at least one chick had to be fully visible to the camera to be included in the dataset.

We recorded the duration of parental behaviors (see Table 1) by scoring the start and end time of each behavior, and used these numbers to calculate duration. In all cases we recorded the sex of the individual engaged in the behavior. Additionally, we recorded the number of nest disturbances by people or other animals per day, and used an ordinal scale of 0–10 (with 10 being the highest and equivalent to something entering the falcon’s nest) to measure the level of each disturbance to the nesting falcons (Table S1). The disturbances were considered to be additive per day; for example, if a nest was entered two times in one day, the disturbance level for the day would be equal to 20.

**Table 1:** Parental behavior recorded at each falcon nest.

Behavior	Description	Data obtained for analysis
Nest attendance	Time spent by adults in the nest, including being engaged in all of the behaviorsbelow, as well as when in the nest, but not touching chicks or engagingin other defined behavior.	Proportion of the daily total(s).
Nest activity	Number of times adult falcons departed the nest; used as a proxy for activityat the nest entrance (see [25]).	Counts.
Brooding	Adult falcon is physically touching at least one chick with breast, tail, or wings.Also applies if falcon is standing over chicks to provide shade(stress brooding).	Proportion of the daily total(s). Count of brooding bouts. Average length of brooding bouts.
Nest maintenance	Adult falcon is pulling at substrate within scrape. Also applies to removingitems such as prey remains.	Proportion of the daily total(s).
Feeding	Adult falcon is feeding food to chicks or is eating.	Proportion of the daily total(s). Counts of feeding events. Average time(s) between feeding events. Average biomass (g) of individual prey items. Sum of prey biomass (g)

Over half of the prey items delivered to the nest could not be identified to species and we estimated the biomass of these items by comparing the size of the prey item with previous, positively identified prey items. The one-day-old poultry chicks (c. 40 g) provided as supplementary food were larger than the finch and bunting species commonly consumed by falcons [Kross SM, Tylianakis JM, Nelson XJ unpublished manuscript] and, because they were easily identifiable due to their bright yellow color, all were identified when they were delivered to chicks. We collected information on the amount of prey handling that occurred prior to items being delivered to the nest by the parents. Avian prey were aged according to feather structure: birds with completely sheathed feathers were considered nestlings, those with partially sheathed feathers were considered fledglings, and those with unsheathed feathers were considered adults [29]. The amount of prey handling done prior to parents delivering the item to chicks was noted, with prey being either completely plucked (no wing or tail feathers remaining), partially plucked (some wing or tail feathers remaining) or not plucked (all wing and tail feathers intact). We also noted the presence or absence of the preys’ head at the time of delivery to the nest.

### Data Analysis

Data from individual nests were analyzed with increasing chick age in days as a predictor variable, defined using the hatching date as chick age 0. In order to maximize data collection for all chicks, data were collected until day 30; the age at which chicks begin to fledge from the nest [23]. Daily data recording began at 05∶00 and ended at 21∶00. These times were chosen because feeding events never occurred prior to 5 am, and out of a total of 2026 feeding events recorded, only 11 occurred after 9 pm (i.e., 99.5% of feeding events occurred during these hours).

We examined parental time budgets by calculating the proportion of the recorded daylight hours adult falcons spent feeding chicks, in attendance at the nest, brooding chicks, or performing nest maintenance. These data were then transformed using a logit transformation [30], and modeled using generalized linear mixed effects models (GLMMs) with Gaussian errors in the lme4 package [31] in R (v.2.7.2) [32]. We were unable to use binomial errors because our proportion time data were not derived from proportions of successes/failures in a fixed number of independent binary trials. Separate models were analyzed for male and female adult falcons, and for both parents combined. The average time between feeding events, the average biomass of prey items, and the average total biomass fed to chicks per day were all modeled using GLMMs with Gaussian errors.

Counts for the amount of nest activity (occasions where parents left the nest), the number of feeding events, and the level of disturbances per day were all modeled using GLMMs with Poisson errors. Feeding data were first analyzed including items identified as supplementary food, and then were analyzed excluding items identified as supplementary food.

Site (i.e. nest identity), the identity of the female and the identity of the male parent were fitted as random effects in all GLMMs. The identity of the parents was included as a random effect to control for non-independence of data between nests containing the same individual male or female falcon (across years, no two nests contained the same pair of adult falcons, but in a few cases either a male or female was paired with a different mate at a different nest site location). We included habitat type, the number of chicks in the nest, and level of disturbances as categorical fixed effects in the models. Chick age in days was included as a continuous fixed effect in the models. We also included an interaction term between chick age and habitat type, as well as quadratic and cubic polynomial terms for chick age in the models to account for potential nonlinear effects of chick age (e.g., asymptotes or step-changes in behavior once a threshold age is reached).

Models were simplified by sequentially removing non-significant polynomial and interaction terms then main effects until no improvement in model fit (measured using the Akaike Information Criterion, AIC) was obtained. We tested all Poisson models for evidence of overdispersion (on the basis of the ratio of residual deviance to degrees of freedom) and re-fitted overdispersed models using penalized quasi likelihood (the ‘glmmPQL’ function) in the MASS package [33] in R. For models fitted using Gaussian errors we used a Markov chain Monte Carlo (MCMC) resampling method with 10,000 simulations to estimate *P* values for the fixed effects (carried out using the ‘pvals.fnc’ function in the languageR package [34] in R). We used Student’s t-tests to compare the prey handling behavior and the age classes of prey for falcons in the two habitats, as well as to compare the number and level of disturbances by people or animals at the nests. In our results, where relevant, we present the mean (± SD) for untransformed data (as a measure of effect size) in addition to *P* and ± SE values from model estimates.

## Results

### Feeding Behavior

The number and level of nest disturbances by people or animals did not differ significantly between the two habitats (*t* = −0.51, *P* = 0.63). In vineyard nests, supplementary food items represented 17.89%±8.94% of prey items adults provisioned to their chicks. At three of the vineyard nests, supplementary food items represented <10% of the prey items brought to chicks. However, at the fourth nest, supplementary food items represented 44.53% of prey items brought to chicks.

**Figure 1:**
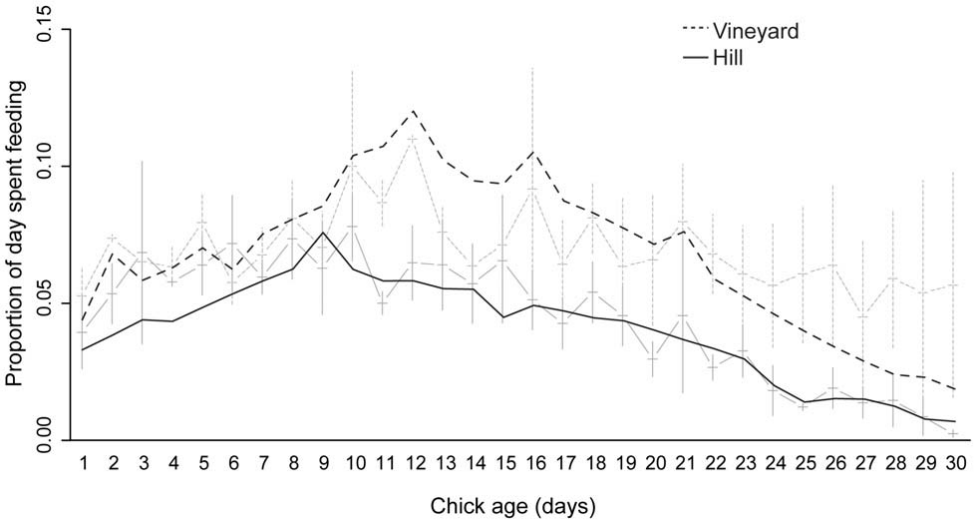
Proportion of the day that parents spent feeding chicks in vineyard and hill nests. Dark lines are the fitted model estimates from a GLMM with a second-order polynomial fitted for chick age. Pale lines are raw data (+/− SEM). Falcons in vineyard nests spent a significantly greater proportion of the day feeding chicks compared with falcons in hill nests (*P*<0.05).

Falcons from nests in the hills spent a significantly lower proportion of their time feeding chicks than did falcons nesting in vineyards (Table 2; Figure 1). Feeding decreased as chicks aged, although more so in hill nests than in vineyard nests (habitat x chick age interaction: Table 2; Figure 1). In hill nests, parents increased the proportion of the day spent feeding from chick hatching until chicks were approximately 9 days old, after which they began to decrease. In vineyard nests, this switch occurred later, when chicks were approximately 12 days old (quadratic polynomial term; Table 2; Figure 1).

**Table 2:** Generalized linear mixed effects model coefficients for feeding parameters measured for breeding New Zealand falcons *Falco novaeseelandiae* nesting in managed vineyards and unmanaged hill habitat.

Response variable		Intercept	Effect of habitat (hill nests)	Effect of chick age (per day)	Effect of interactionbetween habitatand chick age	2 chicks inbrood	3 chicks inbrood	Quadratic polynomial term	Cubic polynomial term
Proportion of day spentfeeding chicks	Estimate	0.15	0.08 (−0.06)	0.15 (−0.005)	R	R	R	0.15 (<0.001)	R
	t-value	−6.64	−2.12	−6.82				−7.97	
	p-value	<0.001	0.04	<0.001				<0.001	
Interval between feedingbouts (sec)	Estimate	6339.36	7180.40 (841.04)	6313.80 (−25.56)	6309.36 (−29.532)	4363.34 (−1976.03)	3273.99 (−3065.37)	6348.48 (9.119)	6339.05 (−0.311)
	t-value	9.554	1.313	−0.807	−1.132	−2.346	−6.992	5.815	−1.457
	p-value	0.0010	0.2314	0.4376	0.2612	0.0418	0.0001	0.0001	0.1660
Number of feeding eventsper day by female	Estimate	8.89	9.44 (0.55)	8.99 (0.10)	8.79 (−0.10)	13.23 (4.39)	12.81 (3.92)	8.87 (−0.02)	R
	t-value	19.01	0.43	2.64	−3.33	2.77	5.00	−5.93	
	p-value	<0.001	0.67	0.008	<0.001	0.006	<0.001	<0.001	
Number of feeding eventsper day by male	Estimate	0.036	R	0.041 (0.005)	R	0.08 (0.04)	0.15 (0.11)	0.035 (−0.001)	R
	t-value	−7.88		7.58		1.21	4.27	−2.20	
	p-value	<0.001		<0.001		0.23	<0.001	0.03	
Number of feedingevents by both parents	Estimate	7.00	7.12 (0.12)	R	R	9.76 (2.76)	11.04 (4.04)	6.99 (−0.01)	R
	t-value	23.76	5.26			2.77	6.62	−4.47	
	p-value	<0.001	<0.001			0.006	<0.001	<0.001	
Number of feedingevents withoutsupplementary food	*Estimate*	*5.94*	*7.35 (1.41)*	*6.01 (0.07)*	*R*	*7.51 (1.57)*	*7.92 (1.98)*	*R*	*R*
	*t-value*	*20.85*	*3.48*	*3.84*		*2.44*	*4.73*		
	*p-value*	*<0.001*	*<0.001*	*<0.001*		*0.01*	*<0.001*		
Mean biomass ofprey items	Estimate	20.00	23.56 (3.56)	20.28 (0.28)	19.89 (−0.10)	R	R	R	R
	t-value	7.97	1.08	2.96	−2.70				
	p-value	<0.001	0.29	0.007	0.011				
Mean biomass ofprey items (supplementaryfood removed)	*Estimate*	*17.98*	*23.58 (5.60)*	*18.28 (0.30)*	*17.88 (*−*0.10)*	*R*	*R*	*R*	*R*
	*t-value*	*7.04*	*1.66*	*3.11*	−*2.78*				
	*p-value*	*<0.001*	*0.11*	*0.003*	*0.006*				
Total daily biomass	Estimate	101.59	118.51 (16.91)	109.18 (7.58)	104.01 (2.42)	256.04 (154.95)	250.55 (148.95)	101.06 (−0.53)	R
	t-value	2.71	0.38	8.41	−3.82	2.44	5.35	−6.62	
	p-value	0.011	0.72	<0.001	<0.001	0.18	<0.001	<0.001	
Total daily biomass without supplementary food	*Estimate*	*87.69*	*155.66 (67.97)*	*92.51 (4.82)*	*89.88 (2.19)*	*197.14 (109.45)*	*168.22 (80.54)*	*87.30 (*−*0.38)*	*R*
	*t-value*	*2.95*	*1.92*	*4.98*	−*1.82*	*2.53*	*3.93*	−*4.41*	
	*p-value*	*0.016*	*0.083*	*<0.001*	*0.076*	*0.031*	*<0.001*	*<0.001*	

Models for count data were analyzed using Poisson errors, and all other data were logit transformed and modelled using Gaussian errors. Supplementary food items were provided to falcons nesting in vineyards on a daily basis and weighed an average of 40 g. Models for data where supplementary food items have been removed are shown in italics. Values shown in parentheses under estimates show the change from the model intercept. R =  Predictor variable removed during model simplification.

Vineyard and hill nests did not differ significantly in the interval between feeding bouts (Table 2). Regardless of habitat type or chick age, nests containing a greater number of chicks experienced less time between feeding bouts (Table 2). The number of feeding events per day was also influenced by habitat type and chick age. In both habitats, as chick age increased, male falcons delivered more food items to the chicks, starting at an average of 0.03 feedings per day when chicks first hatched and increasing by 0.04 feedings for each day as chicks aged (Table 2). Female falcons in vineyard nests also increased their number of feeding events as chick age increased, starting at an average of 8.89 feedings per day when chicks first hatched and increasing by 0.10 feedings per day as chick age increased (Table 2). In contrast, females in hill nests started at an average of 9.44 feeding events per day when chicks first hatched, but decreased the number of feedings by 0.20 per day as chick age increased (Table 2). When supplementary food was excluded from the analysis, nests in the hills had an average of 1.41 more feeding events per day (Table 2) compared with vineyard nests. Removing supplementary food from the analysis did not change the fact that, compared with nests with one chick, nests containing 2 chicks and 3 chicks received more food (2.44 and 4.73 more feeding events per day, respectively; Table 2). The quadratic polynomial for chick age was retained in the final model for the number of feeding events, suggesting a nonlinear relationship, but was removed from the final model excluding supplementary food, suggesting a linear relationship.

At the time of hatching, there was no effect of habitat type (Table 2) on the average biomass of each individual prey item consumed by chicks (hill, 23.56±3.31 g; vineyard, 20.00±2.51 g). However, as chick age increased, the average biomass of prey items in vineyard nests increased, while the average biomass of prey items in hill nests decreased slightly (chick age x habitat interaction, Table 2). Excluding supplementary food (mean biomass of a day-old poultry chick was 40 g) from this analysis reduced the average biomass slightly in vineyard nests (17.98±2.55 g) at the time of hatching, but there remained no significant effect of habitat type in our model (Table 2). Even with supplementary food excluded from the analysis, the average biomass of prey items increased in vineyard nests, but decreased in hill nests (chick age x habitat interaction, Table 2).

**Figure 2:**
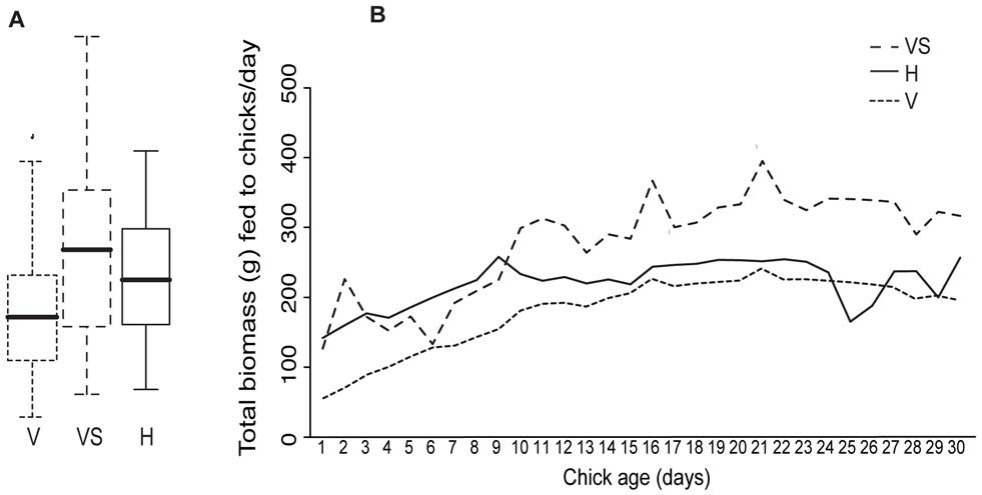
The total biomass of prey brought into nests in vineyards and hills. **A** The minimum, lower quartile, median, upper quartile, and maximum observations for vineyard nests with supplementary food items excluded (V), for vineyard nests including supplementary food items (VS) and for hill nests (H). **B** The fitted model estimates from a GLMM with a significant second order polynomial fitted for chick age, including supplementary food for vineyard nests (VS) and excluding supplementary food (V) and for hill nests (H). Model estimates indicated that as chick age increased falcons in vineyard nests brought in more total prey each day than did falcons in hill nests (*P*<0.001).

The total biomass of prey fed to chicks each day was the sum of all prey items. When chicks first hatched there was no statistically significant difference in the total biomass fed to them in the different habitat types, but as chicks became older, there was an increasing difference between hill and vineyard nests, with vineyard nest parents feeding chicks an additional 7.58 g per day (chick age effect: Table 2), while parents from the hill nests only fed an additional 2.42 g per day (habitat x chick age interaction: Table 2; Figure 2). Nests with more chicks were also given more food. Keeping all other variables constant, nests with 1 chick received a daily mean ± SEM of 101.59±37.54 g, those with 2 chicks 256.04±63.25 g, while those with 3 chicks received 250.55±27.84 g of food (Table 2). Excluding supplementary food items from the analysis for total biomass reduced the overall estimates for biomass fed to chicks, but did not change the lack of statistically significant differences between habitat types (Table 2). Excluding supplementary food resulted in a non-significant relationship between habitat type and chick age (Table 2). Disregarding supplementary food did not change the positive effect of chick age, or number of chicks in the nest on total biomass, but did slightly reduce the scale of these estimates (Table 2).

Prey handling (i.e. whether the parents had plucked the feathers or fur from their prey or decapitated their prey) was influenced by habitat. A greater proportion of the bird prey delivered to vineyard nests was completely plucked (70.38±2.97%) compared with hill nests (56.10±3.92%, *t = *2.90, *P = *0.02). Hill falcons brought their chicks a greater proportion of partially plucked (21.48±2.88%) and unplucked (17.08±4.54%) avian prey compared with vineyard falcons (15.67±2.96% and 12.32±2.41% respectively) although these differences were not statistically significant (partially plucked: *t = *1.41, *P = *0.2; not plucked: *t* = 0.92, *P* = 0.4). Falcons in vineyard nests decapitated more of the prey items delivered to chicks (68.59±3.29%) than falcons in hill nests (56.31±2.22%, *t* = 3.10, *P* = 0.02).

Only 42.45% of prey items delivered to nests were identified to age class. The diet of falcons in vineyards consisted of a higher proportion of juvenile avian prey (vineyard mean =  5.19±1.94%, hill mean =  1.28±0.73%, *t = *3.86, *P = *0.02), but the two habitats were similar in the proportion of adult (mean =  27.98±11.27%, *P*>0.30) and nestling (mean = 10.91±5.23%, *P*>0.80) prey items in the diets fed to chicks.

### Chick-rearing Behavior

**Figure 3:**
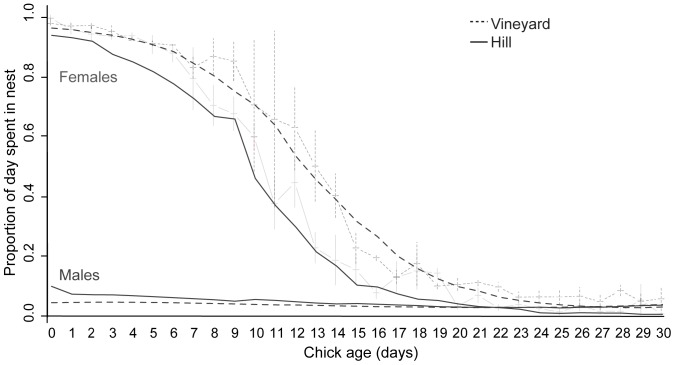
Proportion of the day that both adult falcons were in attendance at the nest as chick age increased in vineyard (dotted lines) and hill (solid lines) nests. Thin grey lines show the raw data for both parents combined, with +SEM for vineyard nests and the data mean –SEM for hill nests. Thick lines show the fitted values from a GLMM including significant second and third order polynomial terms for female falcons (black lines) and from a GLMM including significant second order polynomial terms for male falcons (dark grey lines).

Nest attendance, the proportion of the day that at least one adult was present within the nest scrape (Table 3), was 3.3% lower for parents in hill nests than in vineyard nests (Table 3, Figure 3) and significantly decreased as chicks aged in both habitat types (Table 3, Figure 3). This relationship with age was nonlinear, with the rate of this decline tending to slow after chicks reached approximately 20 days old, and both polynomial terms for chick age were retained in the simplified model (Table 3, Figure 3). This effect was largely due to the behavior of female parents, which were responsible for the majority of nest attendance over the chick-rearing period (Table 3, Figure 3).

**Table 3:** Generalized linear mixed effects model coefficients for parental care parameters measured for breeding New Zealand falcons *Falco novaeseelandiae* nesting in managed vineyards and unmanaged hill habitat.

Response variable		Intercept	Effect of habitat (hill nests)	Effect of chick age (per day)	Effect of interaction between habitat and chick age	2 chicks inbrood	3 chicks inbrood	Quadraticpolynomial	Cubicpolynomial
Nest attendance(both parents)	Estimate (change from intercept)	0.979	0.946 (−0.033)	0.971 (−0.008)	R	0.992 (0.013)	0.983 (0.004)	0.979 (0.0001)	0.979 (<0.001)
	t-value	12.489	−6.003	−18.516		3.646	1.61	6.062	3.092
	p-value	<0.001	0.005	<0.001		0.027	0.160	<0.001	0.003
Nest attendance (females)	Estimate	0.981	0.953 (−0.03)	0.974 (−0.007)	R	R	R	0.981 (<0.001)	0.981 (<0.001)
	t-value	10.4	−3.006	−17.153				3.014	3.703
	p-value	0.0001	0.02	0.0001				0.004	<0.001
Nest attendance (males)	Estimate	0.0524	0.0858	0.0505	0.0492	R	R	0.0525	0.0524
	t-value	−10.920	1.849	−4.454	−4.056			3.217	1.776
	p-value	<0.001	0.031	<0.001	<0.001			<0.001	0.090
Nest activity (both parents)	Estimate	33.857	26.051	33.046	R	R	R	33.819	R
	z-value	56.30	−4.13	−10.83				−4.34	
	p-value	<0.001	<0.001	<0.001				<0.001	
Brooding rates (both parents)	Estimate	0.9370	R	0.9151 (−0.022)	R	R	R	0.9375 (0.005)	0.9371 (<0.001)
	t-value	10.6		−21.85				10.67	6.21
	p-value	<0.001		<0.001				0.001	0.001
Nest maintenance (bothparents)	Estimate	0.0578	R	0.0556 (−0.002)	R	R	R	0.0578	0.0578
	t-value	−32.270		−7.480				2.390	2.740
	p-value	0.0001		0.0001				0.018	0.007

Models for count data were analyzed using Poisson errors, and all other data were logit transformed and modelled using Gaussian errors. Values shown in parentheses under estimates show the change from the model intercept. R =  Predictor variable removed during model simplification.

There was no effect of habitat on the time parents spent brooding chicks. Immediately after hatching, parents in both habitats spent 93.70% of the day brooding (Table 3). In both habitat types, adults significantly decreased the proportion of time spent brooding as chicks aged (Table 3), although this effect was nonlinear, with the slope of the decline leveling out at close to zero once chicks reached approximately 18 days old (quadratic and cubic polynomials Table 3).

The amount of nest activity (the number of times parents left the nest) at vineyard nests was significantly higher than at hill nests, with parents at vineyard nests leaving the nest more frequently (21.84±8.12 daily nest exits) than in hill nests (17.10±6.01 daily exits; Table 3). When chicks first hatched, parents in vineyard nests averaged 33.6 nest exits/day, while those in hill nests averaged 26.1 nest exits/day. However, as chick age increased, parents in both habitats significantly decreased activity around the nest (Table 3), particularly after chicks reached approximately 11 days old (second order polynomial for chick age, Table 3).

There was no effect of habitat on the small proportion of the time per day spent maintaining nests (hill, 0.01±0.02; vineyard, 0.01±0.01) and the habitat term was removed from the simplified model. In both habitat types, adults significantly decreased the time spent maintaining nests as chicks aged (Table 3).

## Discussion

Reintroducing the New Zealand falcon into the vineyards of Marlborough has previously been shown to successfully provide vineyards with a natural form of pest control, by reducing the abundance of pest birds (starlings *Sturnus vulgaris;* song thrushes *Turdus philomelos*; and blackbirds *Turdus merula*) and the amount of damage found on vineyard grapes [22]. However, without evidence of a benefit to the falcons themselves, the effort and cost of translocating individuals of this threatened species to vineyards may be unjustified. Our results show that, within an intensive agricultural area, falcons are capable of feeding their chicks more often and with larger food items, and of spending more time in attendance at the nest, both of which are factors that are associated with increased nesting success [24]–[25].

In addition to spending more time attending and feeding their chicks, vineyard falcons provided better quality food. They provided significantly more plucked and decapitated prey to their nestlings. By completely removing these indigestible food parts, parents provide chicks with food items that are more energy efficient to digest, and that potentially reduce the risk of ectoparasite exposure to chicks [35]. This behavior may also reduce the chances of attracting predators to the nest by avoiding a buildup of prey remains around the nest area [35].

While the differences observed between habitats in this study may have been due in part to the supplementary food provided to the falcons living in vineyards, removing these feeding events from our models still indicates that falcons living in vineyards are at least as good, if not better, at provisioning nestlings with food as those in the hills. Furthermore, removing the supplementary food from our analysis revealed that falcons in vineyards tend to increase the size of average prey items as chick age increases, whereas those in the hills actually catch smaller prey. Therefore, removing these data provides a highly conservative estimate of differences between the habitat types, as vineyard falcons would likely find other food if supplementary food was unavailable. Further experimentation into the effect of supplementary food on falcons in the vineyards will provide the link necessary to distinguish the quality of the two habitats for falcons. Our results provide evidence that New Zealand falcons are capable of displaying the behavioral plasticity necessary to survive and rear their offspring in a highly altered anthropogenic landscape. This concurs with recent results that suggest that this species is capable of nesting in *Pinus radiata* plantation forestry [27], whereas forestry habitat was previously thought to be deleterious to the falcon [23].

Reproduction is an energetically costly phase in the annual cycle of all breeding birds, and a lack of food over any portion of the reproductive cycle can have limiting effects on both parents and their offspring [36]–[37]. Nesting birds of prey must balance the relatively low-cost behaviors of caring for their young in the nest (activities such as brooding) with the need to forage away from the nest - a behavior high in metabolic cost. The availability of prey in the areas surrounding the nest therefore has a direct effect on the breeding success of raptors, as is the case with peregrine falcons, *Falco peregrinus*, where increased nest attendance by females is associated with increased nesting success [25]. Providing supplementary food to altricial birds during breeding can therefore positively affect reproduction rates, fledging condition and parent survival [36], [38]–[39]. Similarly, areas of high prey densities are associated with higher reproductive rates [36], [38]. In our study area, vineyards have a higher density of avian prey compared with hills [Kross SM, Tylianakis JM, Nelson XJ unpublished manuscript], and falcons were additionally provided with supplementary food. It is therefore difficult to tease out the effect of habitat alone, or supplementary food alone, on nesting falcons. While some other raptors (e.g. kestrels, *Falco tinninculus* [40]) have been shown to benefit from supplementary feeding, our results go further, showing that supplementary feeding alone does not fully explain the positive ramifications that we have demonstrated for vineyard habitat.

Females were present within the nest for much more of the day than males. Females therefore took on the majority of the nest-based behaviors that were the focus of this study, and it is likely that males took on the majority of foraging, and provisioned females with prey items with which to feed chicks. This most likely occurs because female falcons, as the physically dominant individual in a pair, remain within or near the nest, and intercept males approaching with food in order to feed the chicks themselves, especially prior to chicks being able to thermoregulate, a pattern that has been shown in the peregrine falcon [41]. If males were unable to forage efficiently and females were forced to forage in order to provision chicks, especially when chicks were not yet able to thermoregulate, this could result in lower nesting success. In our study, supplementary food was only relied upon as a food source by one of the vineyard pairs: the remaining 3 pairs used supplementary food for <10% of their feedings. Interestingly, in these 3 pairs, 98.25% of the supplementary food items were brought to the nest after chicks had reached 14 days of age, by which time adult females had drastically reduced the amount of time they spent in the nest (Figure 3) and were likely to have joined their mates in foraging and food provisioning. Male kestrels have been shown to avoid provisioning their chicks with supplementary food items, whereas females feed both themselves and their chicks with supplementary food when it is available [40], and our results indicate that it is possible this is also the case in New Zealand falcon. Experimentally providing only some of the vineyard falcons with supplementary food in the future will lead to further understanding of the effect of habitat alone in the breeding behavior of the threatened New Zealand falcon.

Parents in nests with more chicks fed their chicks a greater total biomass per day, and fed them more often. However, these increases did not fully compensate for the sharing of food items amongst chicks. On average, single chicks received more food per day (174 g), than each of two chicks (131 g) or three chicks (97 g), and this effect remained even after removing supplementary food from the analysis. These results indicate that removing chicks from hill nests (as carried out by the FFG project) may benefit the remaining chick through increased food provisioning. However, this assumption does not take into account the behavioral impact of removing siblings on the remaining chick [42], or the impact of this harvest of individuals on the falcon population in the hills [18].

One important caveat to the conservation implications of this study is mortality as a consequence of electrocution, which may increase due to the prevalence of power lines in anthropogenic habitats. There is some evidence [43] to suggest that falcons residing in vineyards are suffering significant losses due to electrocution, a common pattern among raptors [5]. However, it has recently been demonstrated that if political will can be found, initiatives to mitigate these effects are both effective and affordable [7].

Our results suggest that there is considerable potential in the idea of reintroducing falcons into vineyards. We have previously demonstrated significant economic benefits for vineyards containing falcons due to a reduction in damaged or destroyed grapes [22]. Here, we showed that there may also be beneficial effects for falcons breeding within vineyards, as falcons in vineyards had higher nest attendance, spent more time feeding chicks, and fed chicks more often and with more food compared with falcons in hill nests.

## Supporting Information

Table S1 Explanation of nest disturbance scores.
(DOC)
