# Supplementary material for: Translocation of Threatened New Zealand Falcons to Vineyards Increases Nest Attendance, Brooding and Feeding Rates
Source: PLoS One. 2012 Jun 14;7(6):e38679. doi: 10.1371/journal.pone.0038679 (PMC3375302; doi:10.1371/journal.pone.0038679)
Supplement: Table S1 — Explanation of nest disturbance scores. (DOC) [file pone.0038679.s001.doc]

Supporting Information

Table S1- Explanation of nest disturbance scores

| Nest Disturbance Score | Explanation of meaning |
| --- | --- |
| 1 | No disturbance |
| 2 | A falcon was seen examining the remote videography system |
| 3 | A falcon pulled or pecked at the remote videography system |
| 4 | A falcon was heard kekking* but there were no people, animals or vehicles seen within 500m of the nest |
| 5 | An animal, person or vehicle came within 500 and 200m of the nest |
| 6 | An animal, person or vehicle came between 200 and 100m from the nest |
| 7 | An animal, person or vehicle came between 100 and 50m from the nest |
| 8 | An animal, person or vehicle came between 50 and 25m from the nest scrape |
| 9 | An animal, person or vehicle came within 25m of the nest scrape |
| 10 | The nest scrape was entered by an animal or person |

We used an ordinal scale to measure the level of each visible disturbance to the nesting falcons. In some cases, the angle of the video allowed us to see some of the area outside of the nest scrape. Many of the disturbances recorded were measured on days when the camera system was checked and therefore an observer was in the area of the nest to note falcon, animal and human activities. All remote videography systems were maintained at the same rate.

*Kekking is the aggressive call of the falcon.
